# Nanotherapeutics Plus Immunotherapy in Oncology: Who Brings What to the Table?

**DOI:** 10.3390/pharmaceutics14112326

**Published:** 2022-10-28

**Authors:** Elise Timon-David, Carla Perez, Anne Rodallec

**Affiliations:** COMPO-SMARTc CRCM, UMR Inserm 1068, CNRS UMR 7258, Aix Marseille Université U105, Institut Paoli Calmettes Inria Centre de Recherche Sophia Méditerranée, 13007 Marseille, France

**Keywords:** nanotherapeutics, immunotherapy, oncology, combination

## Abstract

While the number of oncology-related nanotherapeutics and immunotherapies is constantly increasing, cancer patients still suffer from a lack of efficacy and treatment resistance. Among the investigated strategies, patient selection and combinations appear to be of great hope. This review will focus on combining nanotherapeutics and immunotherapies together, how they can dually optimize each other to face such limits, bringing us into a new field called nano-immunotherapy. While looking at current clinical trials, we will expose how passive immunotherapies, such as antibodies and ADCs, can boost nanoparticle tumor uptake and tumor cell internalization. Conversely, we will study how immunotherapies can benefit from nanotherapeutics which can optimize their lipophilicity, permeability, and distribution (e.g., greater tumor uptake, BBB crossing, etc.), tumor, tumor microenvironment, and immune system targeting properties.

## 1. Introduction

As precision medicine is a great hope for cancer patients, targeted therapies such as nanotherapeutics and immunotherapies are considered to be promising options to overcome the complexity of tumor biology and immune desert [1]. Indeed, because of improved pharmacokinetics (e.g., longer half-life, greater tumor uptake), nanoparticles can deliver higher drug concentrations to the tumor site while limiting the accumulation in healthy tissue [2] and biotherapies, such as monoclonal antibodies or more recent immune checkpoint inhibitors, address new therapeutic targets (e.g., HER2, EGFR, CTLA-4, PD-1, and PD-L1) which is essential for a better cure and long-term survival [1,3].

However, recent results showed that each of these therapies is facing a variety of limitations. While nanomedicine fails in clinical trials because of a lack of efficacy [4,5], immunotherapies are restricted to patients whose tumors exhibit specific molecular features [6,7]. Thus, it was shown that the wide heterogeneity of tumors (e.g., vascularization, receptor expression, microenvironment, etc.) and immune systems (e.g., poor T cell response, low immunogenicity, etc.) largely affect these treatments’ efficacy [2,6]. Consequently, combining with radiation therapy, hormonotherapy, or chemotherapy could limit these resistances [8,9].

While the interest in combining antibodies with chemotherapies is widely known [10], the rationale for a chemotherapy/immunotherapy combo is to disrupt the equilibrium between tumor and immune system, thus, promoting immune response. For instance, it was demonstrated that some chemotherapies (e.g., as vinca-alkaloids, taxanes, cyclophosphamide, etc.) can induce immunogenic cancer cell death and increase dendritic cell expression [11,12].

Nanoparticle immunogenicity could further stretch these immunomodulating features. In a recent study in pancreatic cancer, Del Re et al. assessed the ability of gemcitabine plus nab-paclitaxel (i.e., a nab-drug type nanoparticle) to increase the level of PD-L1 mRNA expression, suggesting its interest as an immunomodulatory regimen to increase future chances of success for immune checkpoint inhibitors [13].

From immunogenicity to tumor microenvironment targeting, many other studies illustrate how immunotherapies can benefit from nanomedicines. Going over the most recent clinical trials associating these two kinds of therapies, this review will focus on these examples but also on how nanomedicine can, in return, benefit from immunotherapies.

## 2. Nanotherapeutics at a Glance

**Figure 1:**
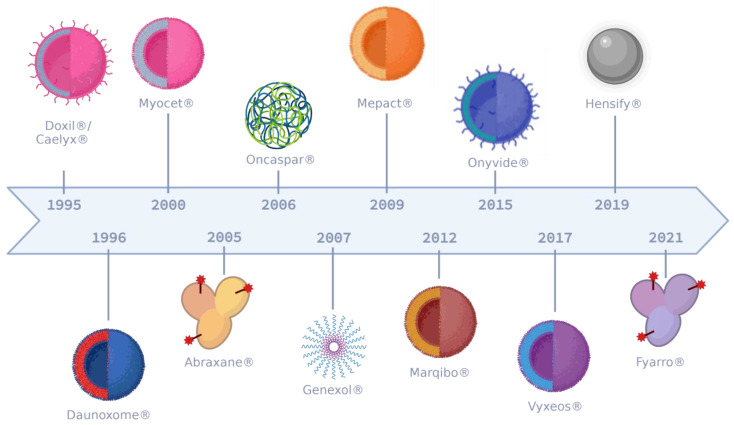
Timeline of the marketing authorization of the keystone cancer nanotherapeutics. In 1995, Doxil®/Caelyx® was the first FDA-approved (pegylated) liposome, followed by unpegylated liposomes; Daunoxome®, Myocet®, Mepact®, Marqibo®, and Vyxeos® and pegylated one: Onyvide®. Vyxeos is the first commercialized nanotherapeutics encapsulating two chemotherapies (i.e., cytarabine and daunorubicin). Other formulations have been approved in the clinic, such as nab-drugs in 2005 and 2021 (i.e., Abraxane® and Fyarro®, respectively), pegylated aparaginase (i.e., Oncaspar®) in 2006, polymeric micelles (i.e., Genexol®) in 2007, and inorganic nanoparticles in 2019 (i.e., Hensify®).

Nanoscale delivery systems or nanotherapeutics are of great interest in oncology [14]. Their characteristics (e.g., size, lipophilic properties, electric charge, PEGylation, carried payloads, etc.) confer them promising pharmacokinetic features such as higher solubility, extended half-life, and optimized distribution among others, promoting a better toxicity–efficacy ratio compared to standard anticancer agents [15]. Since 1964, nanomedicine has made great progress and a plethora of compositions have been developed such as polymeric, lipidic (e.g., liposomes), inorganic (e.g., gold nanoparticles, hafnium oxide nanoparticles), as well as biological (e.g., made from bacteria, virus, cells, etc.) nanoparticles. Among them, polymeric, micellar, and liposomal nanoparticles are the most studied ones [16]. Besides their composition, nanotherapeutics can fulfill different tasks [6,15]: (1) protecting the drug to sustain its circulating time, thus, increasing chances of tumor accumulation and reducing the number of administrations; (2) better targeting of tumors to increase efficacy and reduce toxicities; (3) eliminating excipients from the formulation to reduce excipient-associated premedication and side effects; and (4) harnessing tumor immunity. A few dozen nanotherapeutics can be found on the market (Table 1 and Figure 1), such as liposomal doxorubicin (Doxil^®^/Caelyx^®^) which drastically reduces anthracycline’s cardiotoxicity (i.e., HR = 3.16; 95%CI 1.58–6.31; *p* < 0.001) [17], cremophor-free nab-paclitaxel (Abraxane^®^) which increases survival in pancreatic cancer patients (i.e., median progression free survival from 3.7 to 5.5 months, HR = 0.69 ; 95% CI, 0.58 to 0.82; *p* < 0.001) [18], or liposomal daunorubicin/cytarabine (Vyxeos^®^) which increases survival while reducing the number and duration of infusions (i.e., from 200 mg/m2 over 24 h for 7 days to 100 mg/m^2^ over 90 min on day 1, 3, and 5) [19,20].

Because of their size (i.e., 10–50 nm), antibody drug conjugates (ADC) can also be classified as nanotherapeutics. However, the use of an antibody to cargo their payload makes them into a hybrid entity that belongs to both: nanotherapeutics and immunotherapies. Thus, we chose not to present them in Table 1 but later in this review (i.e., cf. 3.1 Passive immunotherapies).

## 3. Immunotherapy at a Glance

**Figure 2:**
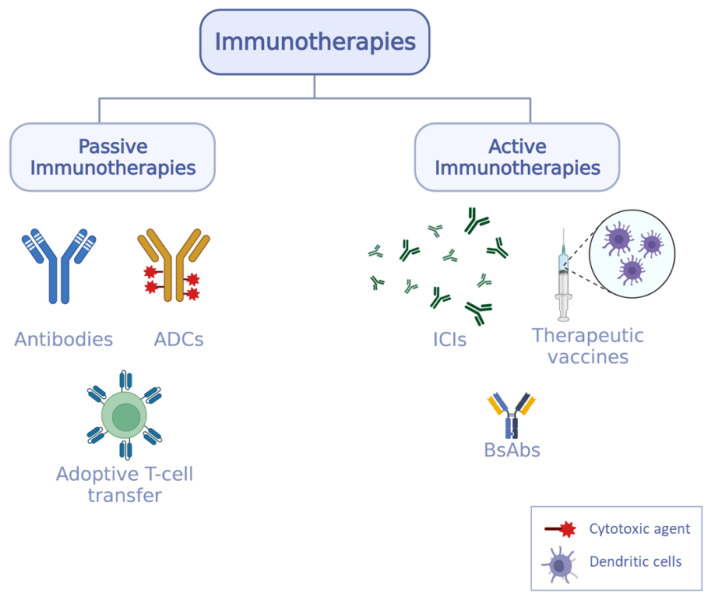
Anticancer immunotherapies classified into passive and active immunotherapies. Passive immunotherapies are the administration of immune molecules directly and active immunotherapies stimulate the patient immune response. ADCs = antibody drug conjugates, T-cell = T lymphocyte, ICIs = immune checkpoint inhibitors, BsAbs = bispecific antibodies.

In oncology, immunotherapies are generally classified into two groups [21]: passive immunotherapy presenting direct antineoplastic activity (i.e., monoclonal antibodies (mAbs), ADCs, and adoptive T-cell transfer) and active immunotherapy (i.e., immune checkpoint inhibitors, bispecific monoclonal antibodies (BsAbs), and therapeutic vaccines) which modulates and stimulates the patient immune system (Figure 2) [22].

### 3.1. Passive Immunotherapies

#### 3.1.1. Monoclonal Antibodies and Antibody Drug Conjugates

The ability of mAbs to specifically bind to an antigen and neutralize it offers patients a new targeted therapy that can be administered in combination with cytotoxics to increase their efficacy [23]. Thus, mAbs have a strong activity on tumors overexpressing their antigens, such as HER2, EGFR, VEGF, or CD20 which led to commercialized molecules that revolutionized the treatment of many solid tumors including lung, colorectal, or breast cancer (Table 2) [24,25].

Although their specific pharmacokinetics (e.g., high molecular weight, low log P) prevent them from being extensively distributed, their antigen specificity makes them promising targeting agents [26]. Consequently, they can be linked to chemotherapy to form an ADC that will spare healthy tissues which do not express the antigen and prevent offsite toxicities. These entities are more selective and achieve higher clinical responses [27], to date 11 ADCs are available in oncology (Table 2); among them, T-DXd (Enhertu^®^) can already be considered as ADC’s next generation since the latest results from phase 3 DESTINY-Breast03 study showed significantly improved progression-free survival in patients with HER2-positive metastatic breast cancer compared to the first commercialized ADC, T-DM1 (Kadcyla^®^) (i.e., HR = 0.28; *p* < 0.001) [28]. In addition, DESTINY-Breast04 trial showed that HER2-low patients could benefit from T-DXd, most probably through a bystander effect of the payload [29].

#### 3.1.2. Adoptive T Cell Transfer

Adoptive T cell transfer represents the last kind of passive immunotherapy, commonly classified as adoptive immunotherapy [30], and consists of reinjecting the patient’s own T lymphocytes after in vitro selection or genetic modification (i.e., CAR-T cells) and expansion for antitumor purposes [31,32,33]. This emerging field has already shown promising results in hematological disorders, and two treatments were approved by the FDA (Table 2). The ultimate objective of such therapies is not only to bring efficient T cells to the patient but also to stimulate and expand their compromised immune system, making it once again into a hybrid therapy that belongs to both passive and active immunotherapy [31].

### 3.2. Active Immunotherapies

#### 3.2.1. Immune Checkpoint Inhibitors and Bispecific Monoclonal Antibodies

Immune checkpoint inhibitors (ICIs) are monoclonal antibodies that regulate lymphocyte T activation by blocking specific ligand/receptor interactions on the cell surface (i.e., tumor cell or T lymphocyte), allowing for antitumor immune response [34]. Among them, anti-CTLA-4 (i.e., ipilimumab) was the first-in-class to be approved against melanoma in 2011. Since then, it has received many more approvals, including in the treatment of lung cancer (Table 3). Later, anti-PD-1 (i.e., nivolumab) was developed and showed longer median progression-free survival alone or combined with anti-CTLA-4 compared to anti-CTLA-4 only (i.e., 11.5 months vs. 6.9 months vs. 2.9 months, respectively) [35]. Similarly, another anti-PD-1, pembrolizumab, was developed and demonstrated prolonged progression-free survival (i.e., 0.46 to 0.72 and 0.47 to 0.72, respectively, HR = 0.58; *p* < 0.001 for both pembrolizumab regimens versus ipilimumab; 95% CI,) with less high-grade toxicity in patients with advanced melanoma [36]. Since then, it is now indicated against many more tumor types (e.g., kidney, NSCLC, head and neck, etc.) (Table 3). Later, other anti-PD-1 treatments were approved, such as sintilimab, camrelizumab, cemiplimab, tislelizumab, and toripalimab [37,38,39,40]. Later on, PD-L1 was discovered as a new target to regulate T reg induction and function [41]. Three anti-PD-L1 ICIs are currently approved: avelumab, durvalumab, and atezolizumab (Table 3). Development of ICIs is constantly evolving, with the emergence of new targets (e.g., Tim-3, NKG2A, TIGIT, etc.) [42] or, more recently, with the approval of a combined ICI solution of anti-PD-1 + anti-TAG-3 for metastatic melanoma. In a phase III trial, this association led to a marked increase in median progression-free survival in untreated melanoma patients with relatlimab–nivolumab as compared with nivolumab (i.e., 10.1 months vs. 4.6 months; HR = 0.75; 95% CI, 0.62 to 0.92]) [43]. Another investigated strategy to enhance T cell recruitment is to combine ICIs with bispecific monoclonal antibodies (BsAbs). BsAbs are antibodies that can simultaneously bind to two different antigen sites [44]. Among them, blinatumomab is the only BsAb FDA-approved in oncology, for the treatment of acute lymphoblastic leukemia. Its dual target CD3/CD19 brings together cytotoxic T-cells and CD-19-overexpressing cancer cells. Recently, its combination with anti-PD-1 pembrolizumab (i.e., NCT03512405, NCT03605589, NCT03340766, and NCT03160079) or nivolumab (i.e., NCT02879695) has been tested in refractory B-cell acute lymphoblastic leukemia patients to overcome blinatumomab resistance. Although the first results of these early clinical phases showed a good tolerance with a high bone marrow percentage [45,46], they recently have been challenged by Giri et al. who demonstrated a lower maximum tolerated dose for blinatumomab in combination with pembrolizumab than for blinatumomab alone, with no efficacy gain [47].

#### 3.2.2. Therapeutic Vaccines

Finally, therapeutic vaccines consist of presenting tumor antigens to guide patient immune systems against neoplastic cells. Different from prophylactic vaccines, they are not intended to prevent a pathology but to turn “cold” tumors into “hot” ones [6,48]. Three vaccination strategies have been developed such as dendritic cell vaccines that are modified ex vivo to sensitize MHC to restricted T lymphocytes, peptide vaccines whose antigens are recognized by T lymphocytes, and genetic vaccines which can administer DNA coding for antigens that will ultimately transfect dendritic cells for similar effects to dendritic vaccines [49]. Only one therapeutic vaccine is currently commercialized. It is a dendritic cell vaccine, Sipuleucel-T, which showed a 22% reduction in the risk of death for prostate cancer patients when compared to the control group (i.e., HR = 0.78; 95% CI, 0.61 to 0.98; *p* = 0.03) (Table 3) [50]. Many other vaccines are currently under study [49].

Although all previously presented therapies (i.e., nanotherapeutics and immunotherapies) have recently taken a significant spot in oncology, they all present caveats in terms of toxicities, efficiency, delivery, and benefiting patients. Nano-immunotherapy is a possible strategy consisting of combining these therapies together to potentiate their benefits while limiting their side effects [51].

## 4. What Immunotherapy Bring to Nanotherapeutics

**Figure 3:**
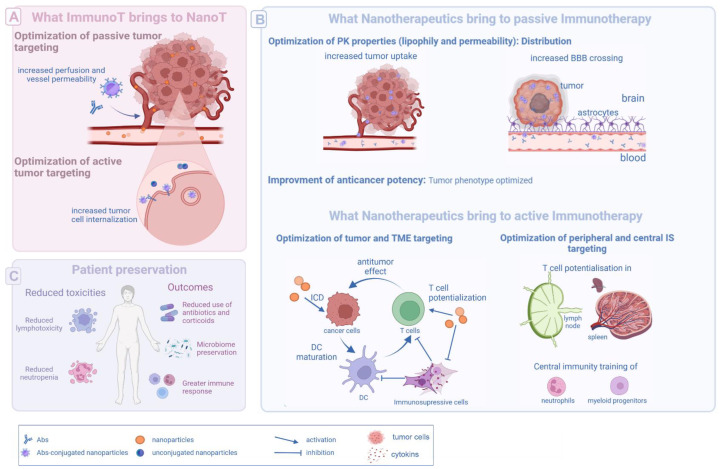
Schematization of the most relevant benefits nanotherapeutics and immunotherapies can get from being combined. Passive immunotherapies (e.g., antibodies) can optimize nanoparticle intratumoral accumulation and internalization (**A**). Nanotherapeutics can optimize passive immunotherapy distributions and modify the tumor phenotype for a better treatment efficacy (**B**). Nanotherapeutics can also optimize active immunotherapy efficacy by targeting the tumor cells, the TME (i.e., T cells or immunosuppressive cells such as MDSCs, Treg, tumor-associated macrophages) or the peripheral/central immune system (**B**). Together, the combination of nanotherapeutics with immunotherapies can protect the patient from poor efficacy, drug toxicity, and associated premedication (**C**). DC = dendritic cell, ICD = immunogenic cell death, IL = interleukin, ImmunoT = immunotherapy, IS = immune system, PK = pharmacokinetics, NanoT = nanotherapeutics, T cell = lymphocyte T, TME = tumor microenvironment, Treg = regulatory T cell, MDSCs = myeloid-derived suppressor cell.

To date, only passive immunotherapies can provide benefits to nanotherapeutics and initiate a synergic effect. It is done through passive and active targeting optimization (Figure 3A).

### 4.1. Optimization of Tumor Passive Targeting

Enhanced permeation and retention effect (EPR) was defined by Maeda as the anarchic vascularization surrounding solid tumors with the absence of lymphatic drainage [52]. Molecules under 200 nm [53,54], such as nanotherapeutics, can accumulate there and avoid healthy tissue distribution. Although techniques to implement the EPR effect on humans are challenging and time consuming [55], it is possible to observe a 25% greater intratumor exposure in patients for docetaxel nanoparticles [56]. However, a recent review declared that this improved accumulation was very heterogeneous and only represented 0.7% of the administered dose, leaving room for optimization [57]. The EPR effect is maximized when the extracellular matrix is low and there is a large amount of blood vessels [58]. Several techniques have, therefore, been studied to reshape tumor vascularization, such as antibodies that can increase tumor perfusion. Sorace et al. demonstrated in HER2+ breast cancer mice that trastuzumab-treated tumors versus control exhibited a significant increase in perfusion and vessel permeability (*p* = 0.035) [59]. Similar results were observed with trastuzumab engrafted on the liposomal surface, which turned poorly vascularized central tumors into highly vascularized ones when compared to ungrafted liposomes [60].

### 4.2. Optimization of Tumor Active Targeting and Cell Internalization

Lately, a strategy that consists of conjugating passive immunotherapies (i.e., antibodies) onto the nanoparticle surface has emerged and shown selective drug delivery to tumor cells with increased tumor cell internalization and cytotoxicity when compared to standard liposomes [61]. Interestingly, mAbs do not necessarily affect tumor uptake. Similar tumor localization was found in mice for trastuzumab–docetaxel immunoliposome and liposomal docetaxel (i.e., 10 ± 1% and 9 ± 1% of administered dose, respectively) [62]. Similar results were observed for immunoliposomes conjugated with anti-HER2 mAb fragments such as Fab or single chain Fv (i.e., 7–8% tumor accumulation of administered dose). Interestingly, Kirpotin’s team were able to demonstrate that engraftment of Abs could indeed not increase tumor uptake but could decrease tumor microenvironment (TME) accumulation (i.e., stroma and macrophages) to the benefit of cancer cells [63]. Similar results were observed for cetuximab-conjugated gold nanoparticles for which greater lung cancer cell internalization was displayed when compared to pegylated gold nanoparticles that remain in the tumor interstitium [64]. Based on tumor antigen overexpression and easy access, other antibodies can be used as targeting agents for nanotherapeutics: anti-EGFR, anti-PSMA, anti-CD20, anti-PD-L1, etc. [65,66]. These targeting agents can simultaneously be used as therapeutic agents and will, in return, benefit from the nanoparticle in the matter of bioavailability, tumor uptake, and systemic exposure [67].

## 5. What Nanotherapeutics Bring to Immunotherapy

### 5.1. Nanotherapeutics to the Rescue of Passive Immunotherapies

As previously mentioned, mAbs present a poor pharmacokinetic with low access to tumors (i.e., tumor concentration from 0.07 to 7% in men and mice) [68]. Thus, they can benefit from nanoparticles whose specific permeability and lipophilic properties give them access to an optimized distribution with higher availability and avoid blood–brain barrier limitations [69]. For instance, Sousa et al. showed that loading bevacizumab into polymeric nanoparticles via the intranasal route significantly increased brain exposure concentration of mAbs for the treatment of glioblastoma (i.e., 5400 ± 2313 ng/g brain tissue vs. 1346 ± 391 ng/g for free bevacizumab, *p* < 0.05) [70]. More recently, the development of a cetuximab-conjugated gold nanoparticle in the colorectal cancer cell, showed greater cytotoxicity versus standard cetuximab, probably because of tumor phenotype modulation by nanomaterials which contributed to upregulation of the anti-EGFR pathway (i.e., EpCAM, CMAM, and HER-3) [71]. Similar results were observed on lymphoma and breast cancer cells with rituximab and trastuzumab liposomes, respectively [72]. Results were confirmed on mice bearing breast tumor xenografts, for which tumor volumes for animals treated with liposomal trastuzumab were significantly lower than for those treated with standard trastuzumab [72]. Such an increase in efficacy can be explained with the optimized pharmacokinetic profile antibodies can gain from liposomal formulation (i.e., slower clearance, larger AUC), which, here, led to an increase in trastuzumab intratumor localization from 3.84 ± 2.4 to 13.9 ± 3.4% of injected dose at 24 h [72]. This modification in mAb pharmacokinetics can then allow for sustained mAb release which would ultimately increase intervals between administration and the patient’s comfort [67].

These examples illustrate a first optimization of immunotherapies with nanotherapeutics, many more exist, for active immunotherapies especially.

### 5.2. Nanotherapeutics to the Rescue of Active Immunotherapies

As previously described by Shi and Lammers, three main strategies have been developed to improve active immunotherapy impact with nanotherapeutics: (1) target cancer cells and induce immunogenic cell death (ICD), (2) target and immunomodulate TME to promote immune-activation, and (3) target peripheral and central immune system to potentiate antigen presentation and activate and train immune cells (Figure 3B) [73,74].

#### 5.2.1. Target Cancer Cells and Induce Immunogenic Cell Death

Immunogenic cell death (ICD) is a specific category of cell death that can be induced with specific chemotherapies [11,12]. It results in tumor antigen release which activates antigen presentation and cytotoxic T cells, triggering antitumor immunity (Figure 3B) [73]. More recently, Zhao et al. demonstrated that encapsulation of oxaliplatin can significantly increase ICD when compared to the same free drug (i.e., increase of specific damage-associated molecular patterns of about 70% and 48% in HMGB1 release and ATP secretion, respectively) [75]. Indeed, stronger immune responses of dendritic cells and T lymphocytes were achieved in vitro, resulting in stronger therapeutic effects in mice [75]. Similar results were observed for 5-FU and doxorubicin [75,76]. To this extent, several nanoparticles have been designed to reinforce ICD inducers such as doxorubicin, epirubicin, paclitaxel, oxaliplatin, and others [77]. Thus, excellent therapeutic effects can be observed when associated with ICI. For instance, anti-PD-1 antibodies showed in mice greater efficacy when associated with a pH-responsive doxorubicin delivery nanosystem that maintained the antitumor activity of ICD-instigated T cells [78]. Similarly, another doxorubicin-loaded nanovesicle displayed immunogenic cell death in melanoma, lung, and breast tumor cancer mice models, with subsequent DC maturation and T-cell activation, leading to a synergic antitumor effect when combined with anti-PD-1 (i.e., significantly prolonged overall survival time with 33.3% of the mice being tumor-free) [79]. Therefore, nanotherapeutics can improve immunotherapy efficacy and impact patients who, until then, did not respond to these therapies. More immunomodulation effects can be observed on surrounding cells: the tumor microenvironment.

#### 5.2.2. Target and Immunomodulate Tumor Microenvironment

Tumors are not the single cell mass once described but are much more complex and actually include blood vessels, immune cells, and associated cytokines that reflect the inflammatory state and tumor response to therapies [80]. An in-depth analysis sorted two classes of TME that can predict immunotherapeutic reactivity of some treatments such as ICI. Thus, unfavorable TME presents a lack of infiltrating T lymphocytes, increases Treg and myeloid-derived suppressive cells (MDSCs), strong stroma, and are called immunologically “cold tumors” [6]. Many studies demonstrated that the immunogenicity of nanotherapeutics can turn them into “hot” ones through several mechanisms ranging from immunosuppressive cell depletion (e.g., MDSCs, Treg, tumor-associated macrophages) to increased T cell activity (Figure 3B) [6]. Alleviating immunosuppression can be achieved with nanotherapeutics targeting immunosuppressive cells but also by modulating levels of specific cytokines (e.g., IDO, TGF-β) responsible for TME cell communication [81]. Some cytokines (e.g., IFN-γ, IL-2), brought or stimulated by nanoparticles, can be responsible for T cell potentiation. Recently, NBTXR3, a hafnium oxide nanoparticle used as a radio-enhancer, was tested in a mouse colorectal model and was found to be responsible for an increase in CD8+ infiltrates inside the tumor when compared to radiotherapy alone [82]. Such results were further confirmed in a murine lung cancer model for which not only CD8 T-cells were increased but Treg cells were downregulated as well [83]. Further immunomodulatory exploration revealed modifications in gene expression associated with T-cell, NK-cell, and macrophage functions (i.e., Gzmb, Cd8a, Itgal, Ccl3, Il1a, Atg5, etc.). Interestingly, when this radio-enhancer was associated with anti-PD1, this resulted in an improvement in survival rate from 0 to 50% when compared to any other treatments [83]. Finally, to promote immunoactivation, nanotherapeutics can also regulate antigen expression (e.g., CTLA-4, PD-1/PD-L1) [6,73] by encapsulating SiRNA or ASO to silence immunosuppressive targets [84]. When associated with chemotherapies, these nanoparticles can result in a 90% reduction in tumor size in mice, leading to a 100% survival rate (i.e., PD-L1 silencing metallic nanoparticles for pancreatic cancer) [84]. Similar results were observed against breast cancer, for anti-CTLA-4 siRNA-loaded nanoparticles, which significantly reduced CTLA-4-expressing T cells in tumor cells but also in the spleen, leading to tumor regression and increased survival for mice [85]. The composition of nanoparticles has a strong impact on their immunomodulation properties; thus, viral nanoparticles can be of high interest. NanoCarrier^®^ developed VB-111, an adenovirus-5 nanoparticle that serves as an immune adjuvant by activating T-cell infiltration in the tumor. VB-111 is currently under clinical trials for ovarian and colorectal cancers (i.e., NCT03398655 and NCT04166383) [86]. Composition was also studied around lipidic nanoparticles in colon carcinoma mouse models and showed that DOTAP/DOPE-based formulation could decrease splenic MDSC population more than other lipids (i.e., DMPC/DMPG, DSPC/DSPG, HSPC/PEG-DSPE), promoting a stronger immune response. Interestingly, a similar result was not found in the tumor, giving way to another major strategy in oncology: targeting the immune system [87].

#### 5.2.3. Target Peripheral and Central Immune System

Lymphoid organs such as lymph nodes or the spleen play an important role in cancer progression and modulation of the cancer immunological microenvironment [88]. By targeting this peripheral immune system, the objective is to restore patient immune function as antigen presentation and T-cell generation take place there (Figure 3B). To promote immunoactivation, nanotherapeutics adopt similar strategies to the ones presented above, including potentiation of lymphocytes T and tumor antigen presentation, by mimicking antigen-presenting cells to form MHC/artificial APC complexes and, subsequently, generate new antitumor lymphocytes T [89]. For instance, nanoparticles can act like an adjuvant for therapeutic vaccines by enhancing antigen availability and prolonging interaction with immune cells into lymph nodes. To this extent, Zeng et al. developed a nanoemulsion that can coencapsulate ovalbumin-Clec-9A-antigen: Clec-9A being responsible for cancer immunity. Such a carrier was then able to induce specific antibody responses in mice and CD4+ and CD8+ T cell proliferation within the tumor, the spleen, and inguinal lymph nodes, resulting in a very effective tumor immunity therapeutic (i.e., 10-fold tumor size reduction after 24 days) [90]. Similarly, this ovalbumin-Clec-9A-antigen nanoemulsion was found to promote MyD88-dependent DC activation and IFN-α production. After IV injection, they observed an increase in CD86, CD80, and CD40 expression by CD8^+^ DCs, CD8^−^ DCs, and pDCs, suggesting an upregulation in DC activation. Such results were only confirmed for the Clec-9A-targeted delivery system underlying its potential immunogenicity properties [90]. Similar results were observed for a three immunoadjuvant-loaded multiantigenic nanoparticle (MANP), which induced DC maturation (i.e., an increase in CD80 and CD86) [91]. Interestingly such results were even more remarkable for small diameter nanoparticles (i.e., 83 nm vs. 122 nm), ultimately resulting in a more effective delivery to lymph nodes and better antigen presentation to T lymphocytes [91]. Nanoparticles can also boost lymphocytes T activity by regulating the immune phenotype to be favorable (e.g., increased levels of strategic cytokines such as TNF-α, IL-6, IL-12 and GM-CSF) [48]; this was also confirmed with MANPs, which largely increase IL-2 and IFN-γ secretion, inducing specific cytotoxic response [91].

More recently, a new strategy has emerged, and a few teams are now focusing on the innate immune system and, thus, on directly targeting myeloid progenitor cells within the bone marrow or the thymus. For instance, Mulder’s team developed a bone-marrow avid biological nanoparticle that efficiently delivers drugs to myeloid cells, resulting in a trained immunity and a decrease in TAMs which inevitably led to a favorable antitumor response as a monotherapy or in association with ICIs [92]. On a similar note, MonTaBioscience^®^ developed a TLR7 agonist micelle (i.e., MBS8) that triggers migration of innate immune cells (i.e., neutrophils) and also adaptive immune cells (i.e., CD8+ T cells) drastically reducing the tumor volume of pancreatic-tumor-bearing mice as a monotherapy or combined with anti-PD-1 [93]. MBS8 safety and preliminary efficacy are currently under phase 1 clinical study (i.e., NCT04855435), involving 69 patients with advanced solid tumors.

Although most of the previously presented strategies seem to focus on promoting ICI efficiency only, it is important to note that “hot tumor phenotype”, targeting properties, and subsequently reduced side effects can be beneficial to all therapies, especially once patients are at stake (Figure 3).

### 5.3. Preservation of the Patient’s Organism and Immune System

Indeed, all these targeting properties indirectly imply the preservation of the patient’s organism and immune system (Figure 3C). It is well-known that anticancer drugs can be responsible for many side effects that can be limited by vectorization. Among them, lymphotoxicity is implicated in poor immune response and deleterious clinical outcomes for patients treated with immunotherapies and can be reduced with nanoformulations [94]. Similarly, neutropenia-related infections can be reduced, and patients no longer need antibiotic preventive treatment that can be responsible for gut microbiome disruption and, consequently, a lower survival rate in immunotherapy-treated patients [6]. Another premedication such as corticoid can be avoided with nanotherapeutics. Paclitaxel, for instance, no longer needs premedication when administered as nab-paclitaxel. Indeed, because of an increased solubility, the excipient of paclitaxel (i.e., Cremophor EL) can be omitted, such as its related hypersensitivity reactions [95]. Thus, decreasing the use of corticoids can increase naïve T cell proliferation and immune response to immunotherapies [96]. Indeed, as Maxwell et al. showed in a flank-tumor-bearing mice model, corticosteroid treatment can be responsible for severe and persistent diminution of peripheral CD4+ and CD8+ T cells resulting in lower efficacy for anti-PD-1 treatment [97].

For the bigger picture and for all immunotherapies given in association with chemotherapies, better tolerance also implies fewer postponed or discontinued treatments, and, thus, a better chance of survival for the patient. Several past and ongoing clinical studies can already confirm the potential of such a combination.

## 6. Nanotherapeutics and Immunotherapies: Current Clinical Trials

**Figure 4:**
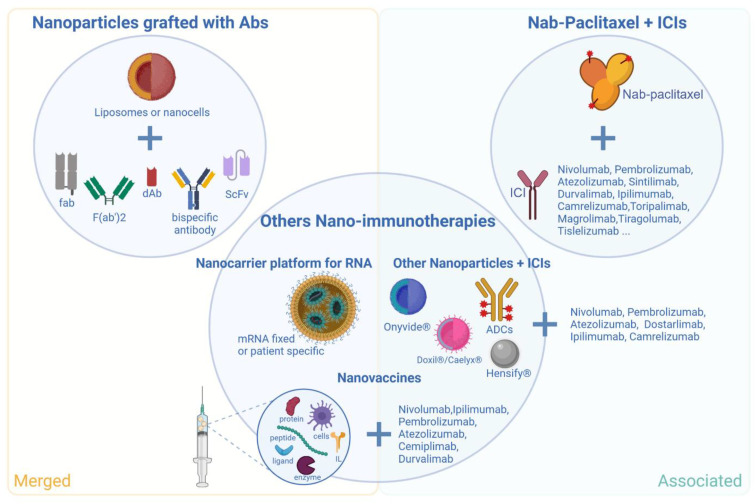
Schematization of the most relevant types of merged (yellow zone) and associated (blue zone) nano-immunotherapies currently under clinical trial in oncology. Merged nano-immunotherapies include nanoparticles (mostly liposomes or nanocells) engrafted with antibodies or fragments of antibodies and nanoparticles used as nanocarrier for immune system activators such as RNA, specific proteins, ligands, enzymes interleukins, or cells. Associated nano-immunotherapies include the combination of ICIs with nab-paclitaxel, nanovaccines, or commercialized nanoparticles (i.e., Onyvide®, Doxil®/Caelyx®, ADCs, Hensify®). ADCs = antibody drug conjugates, dAb = single domain antibody, fab = fragment antigen binding, ICI = immune checkpoint inhibitors, IL = interleukins, scFv = single chain variable fragment.

Most available data on nanotherapeutics + immunotherapies going under clinical trials can be gathered into three groups: (1) nanoparticles grafted with antibodies, (2) nab-paclitaxel (Abraxane^®^) associated with ICIs, and (3) other nano-immunotherapies. Among them, two groups can be dissociated: the combined therapies and the merged ones, for which immunotherapy can directly be linked to or encapsulated in the nanotherapeutics (Figure 4).

### 6.1. Nanoparticles Grafted with Antibodies

As previously mentioned, nanoparticles and passive immunotherapies can both benefit from each other. While nanoparticle targeting and internalization properties can be improved with engraftment of antibodies or fragments of antibodies, the antibodybiodistribution can, in return, be optimized by the nanoparticle lipophilicity. Such symbiosis is developed to improve the therapeutical index by presenting higher efficacy and lower toxicities. In this context, many clinical trials are currently ongoing (Table 4) [98,99,100,101,102]. Among them, nanocells developed by EnGenIC^®^ and liposomes remain the two most studied types of nanotherapeutics. For all the presented early clinical trials, safety of use was demonstrated. Interestingly, MTDs of Ab-conjugated nanoparticles were found to be similar to nonconjugated nanoparticles, except for Erbitux^®^EDVspac, probably because of its bacterial composition [103]. Of note, despite a successful Phase I, some conjugated nanoparticles were never heard from again (e.g., MCC-465), and this combination did not always show benefits. For instance, despite reduced cardiotoxicities, liposomal doxorubicin conjugated to anti-HER2 + trastuzumab was not able to demonstrate any benefit when compared to chemotherapy + trastuzumab [104]. Inadequate study design (e.g., patient selection) could be responsible for this result.

Many other clinical studies have associated nanotherapeutics with unconjugated antibodies, which, for some, this combination was FDA approved. Thus, nab-paclitaxel can be associated with anti-HER2 antibodies (i.e., pertuzumab and trastuzumab) or with anti-PD-L1 ICIs (i.e., atezolizumab) in breast cancer treatment [105].

### 6.2. Nab-Paclitaxel Associated with Immune Checkpoint Inhibitors

Because of large indications and lower toxicities, nab-paclitaxel is one of the most successful nanotherapeutics on the market in oncology. Thus, many clinical trials have been investigating its combination with passive immunotherapies (i.e., bevacizumab, trastuzumab, pertuzumab) but also to ICIs (Table 5). Among them, the results of the IMpassion130 phase 3 trials in advanced-triple-negative-breast cancer stood out [106]. Indeed, for PD-L1 positive tumors, patients receiving nab-paclitaxel + atezolizumab presented a median overall survival of 25 months vs. 15.5 months for patients treated with nab-paclitaxel + placebo (i.e., HR = 0.62; 95% CI) without any new adverse effects [106]. Interestingly, these results were not confirmed in the IMpassion131 phase 3 trial when combining atezolizumab to standard paclitaxel (i.e., HR 1.11, 95% CI; median overall survival 22.1 months with atezolizumab–paclitaxel vs. 28.3 months with placebo–paclitaxel in the PD-L1-positive population), possibly suggesting the importance of a paclitaxel backbone as a nanotherapeutic [107] and nab-paclitaxel’s ability to overcome ICI resistance [108]. This property is, therefore, highly sought after and currently being investigated for unapproved ICIs, such as anti-PD-L1 (i.e., SHR-1701 and ZKAB00), anti-PD-1 (i.e., Tislelizumab), anti-TIGIT (i.e., tiragolumab), and anti-CD47/macrophages (i.e., magrolimab) treatments (Table 5).

For similar reasons, other types of nanoparticles, such as liposomal doxorubicin and irinotecan, have been combined with ICIs. Although, these associations take an important part within the nano-immunotherapies, many other types are currently under clinical study.

### 6.3. Other Nano-Immunotherapies

Based on similar interests, other approved nanotherapeutics are currently being tested in association with ICIs, such as liposomes (i.e., Doxil^®^/Caelyx^®^ and Onyvide^®^) or ADCs (i.e., Blenrep^®^, Adcetris^®^, Enhertu^®^ and Padcev^®^) (Table 6). Indeed, ADCs can present antibody-dependent cellular cytotoxicity (ADCC) with related NK cell activation or ICD with related tumor-infiltrating lymphocyte recruitment, suggesting the possibility of a potentiating effect when combined with immunotherapies [109,110]. For instance, previous results of a phase I clinical study of combined brentuximab vedotin (i.e., Adcetris^®^) with nivolumab in Hodgkin Lymphoma presented a great response and survival rates with limited adverse effects (i.e., overall response rate = 85%, with 67% patients achieving a complete response and progression free survival at 3 years = 77%) [111]. Seeking this opportunity, other unapproved investigational ADCs are also under clinical investigation (e.g., MGC018, Disitamab vedotin, etc.) and paving the way to merged nano-immunotherapies, such as ADCs presenting targeted immunotherapeutic agents (i.e., Mirzotamab clezutoclax, an immunomodulatory targeted, B cell inhibitory ADC under clinical development) [112]. Similarly, inorganic or lipidic nanoparticles are linked to or encapsulate immunotherapies, such as ligands (e.g., OX40), peptides (e.g., survivin, E7), proteins (e.g., GM-CSF and CpG), enzymes (e.g., indoleamine 2,3-dioxygenase), or interleukins (e.g., IL-2, IL-15, IL-23, IL-36) used for tumor-directed immune response (Table 6) [113]. Finally, nanocarriers can also be essential for promising but easily degradable macromolecules, such as RNAs [114]. For instance, BioNTech developed an RNA-lipoplex platform to induce a potent and precise immune response against solid tumors. These entities, also known as nanovaccines, have been under the spotlight for a few years [115] and show high potential. For example, BNT111, a liposomal-RNA vaccine inducing CD4^+^ and CD8^+^ T cell immunity, presented, in a dose-escalation phase I trial, great efficacy with favorable tolerability in 89 advanced melanoma patients, as a single agent or in combination with anti-PD-1 ICIs (i.e., cemiplimab) [116].

## 7. Discussion

Although revolutionary in many respects, nanotherapeutics and immunotherapies remain a challenge for the research and clinical communities. Whereas immunotherapies can lead to complex situations (e.g., unpredictable hypersensitivity reactions, toxicities, etc.), technical issues (e.g., ex vivo preparation of patient immune cells requiring dedicated structures), the use of nanotherapeutics is still limited because of extremely high attrition rates due to several issues with formulation (e.g., size, composition, zeta potential, etc.), scaling, and failure to translate promising experimental results into meaningful efficacy during comparative clinical trials.

In addition to biopharmaceutical synthesis limitations, some challenges remain in the design of clinical trials. Indeed, the many characteristics of nanoparticles (e.g., size, charge, composition, density, etc.) can highly impact their properties (e.g., stability, biocompatibility, drug release, pharmacokinetics, etc.), making it very difficult to predict their behavior [2]. Results in efficacy and toxicity may significantly change from in vitro to in vivo studies and, subsequently, when administered to the patient whose biological components (i.e., protein corona) are much different [117]. For these reasons, and because of the high cost of these promising therapies, new strategies have emerged to efficiently advance and refine these treatments. Among them, patient stratification became obvious, in particular because of previous failed clinical studies (e.g., phase III JAVELIN Ovarian 200 in ovarian cancer or phase II HERMIONE in breast cancer) ([107,118], NCT02213744). Thus, patients could, for instance, be selected based on biomarkers such as receptor expression, tumor perfusion, immunoscoring, and tumor gene expression phenotype [119,120]. Another widely investigated strategy to prevent resistance of nanotherapeutics and immunotherapies is to combine them with other treatments, such as radiation therapy, ultrasound, hyperthermia, chemotherapies, targeted therapies [119,121], and even together initiating a new strategy called nano-immunotherapy.

Indeed, passive immunotherapies such as antibodies and ADCs can boost tumor perfusion, resulting in greater EPR effect and nanoparticle tumor uptake [59,60]. When grafted to the nanoparticle surface, antibodies can also increase the tumor cell internalization of nanoparticles and, therefore, potentialize their antitumor effect [63,64,65]. Similarly, passive immunotherapies can benefit from nanotherapeutics which can optimize their poor pharmacokinetics (e.g., lipophilicity, permeability, and distribution) [69,70,72]. Nanoparticles can also be used as efficient carriers to target the tumor cells, MET, and the peripheral and central IS to immunomodulate the patient’s organism and to optimize the immunotherapy efficacy [73,92,93]. Finally, these targeting properties brought by and for each therapy will subsequently reduce side effects, leading to fewer preventive treatments (e.g., corticoids, and antibiotics), patient preservation (i.e., IS and microbiome preserved), greater immune response, reduction in treatment discontinuation, and greater efficacy [6,94,97].

Two kinds of nano-immunotherapies can be acknowledged: the associated and the merged ones (i.e., when the immunotherapy is grafted, linked, or encapsulated into the nanoparticle). The potential of merging these treatments was studied by Alimohammadi et al. in B16 mouse melanoma models where liposomal anti-CTLA-4 significantly delayed tumor growth when compared to standard anti-CTLA-4 combined with Doxil^®^ (i.e., 113.3%, 22.86%, and 39.04%, respectively) [122]. Interestingly, they also showed that only the administration of anti-CTLA-4 before Doxil^®^ could present synergic efficacy (i.e., tumor growth delay = 161.32% vs. 102.56% and 48.51% for concomitant and Doxil^®^ before anti-CTLA-4 administration, respectively), highlighting the importance of the combination modalities (i.e., duration, sequence, dosing interval, and dose).

A way of predicting such results while avoiding testing infinite in vivo combinations is to develop mathematical models based on drug pharmacokinetics and pharmacodynamics (i.e., PK/PD models) that can simulate and then predict treatment efficacies [15,123]. To this day, only a few models were made available in this field; among them, Cheng et al. constructed a PK/PD model of nanoengineered mesenchymal stem cells in a lung cancer mice model and were able to show that dosing interval had little impact, whereas a higher dose could exhibit greater efficacy [124]. Although the design of clinical trials can be guided with PK/PD modeling, we did not find any within the nano-immunotherapy area, notably because of the recent nature of this field which is reflected in the current advancement of clinical studies.

Indeed, among the 164 clinical trials presented here, only 13 (8%) are in phase III, limiting access to efficacy data. However, considering the large number and preliminary results of these trials, we can only expect that the coming years should be fruitful for nano-immunotherapy and regulatory approval is right around the corner.

## Figures and Tables

**Table 1:** Anticancer nanomedicine currently approved by the FDA and/or EMA. Adapted from Anselmo et al. [5]. Of note: ADCs will be developed in Table 2.

Trade Name	Nanoparticle Type/Drug	Application(s)	Marketing Authorization
Doxil^®^/Caelyx^®^	Liposomal doxorubicin (PEGylated)	Ovarian cancer, kaposi sarcoma, multiple myeloma	FDA (1995) EMA (1996)
DaunoXome^®^	Liposomal daunorubicin	Kaposi sarcoma	FDA (1996)
Myocet^®^	Liposomal doxorubicin	Metastatic breast cancer	EMA (2000)
Abraxane^®^	Nab-paclitaxel	NSCLC, breast cancer, pancreatic cancer	FDA (2005) EMA (2000)
Oncaspar^®^	Polymer protein conjugate	Lymphoblastic leukemia	FDA (2006) EMA (2016)
Genexol^®^	Polymeric micelle	Ovarian, breast, lung, gastric cancers	FDA (2007)
MEPACT^®^	Liposomal mifamurtide	Osteosarcoma	EMA (2009)
Marqibo^®^	Liposomal vincristin	Acute lymphoblastic leukemia	FDA (2012)
Nanotherm^®^	Metallic nanoparticles	Glioblastoma Prostate Cancer	FDA (2010) EMA (2013)
Onivyde^®^	Liposomal irinotecan (PEGylated)	Pancreatic cancer	FDA (2015)
Vyxeos^®^	Liposomal cytarabine/daunorubicin	Acute myeloid leukemia	FDA (2017) EMA (2018)
Hensify^®^	Radio-enhancer crystalline hafnium oxide	Locally advanced squamous cell carcinoma	EMA (2019)
Fyarro^®^	Sirolimus albumin bound nanoparticle	Locally advanced unresectable or metastatic malignant perivascular epithelioid cell tumors	FDA (2021)

**Table 2:** Anticancer passive immunotherapies currently approved by the FDA and/or EMA.

Trade Name	Type of Passive Immunotherapies	Drug/Target	Application(s)	Marketing Authorization
Rituxan^®^	mAbs	Rituximab/CD20	Lymphoma	FDA (1997)
Herceptin^®^	mAbs	Trastuzumab/HER2	Breast cancer	FDA (1998)
Mylotarg^®^	ADC	Gemtuzumab ozogamicine/CD33	Acute myeloid leukemia	FDA (2000 then reapproved 2017) EMA (2018)
Campath-1H^®^	mAbs	Alemtuzumab/CD52	Chronic lymphoid leukemia	FDA (2001)
Erbitux^®^	mAbs	Cetuximab/EGFR	Colorectal cancer	FDA (2004)
Avastin^®^	mAbs	Bevacizumab/VEGF	Colorectal and lung cancer	FDA (2004)
Adcetris^®^	ADC	Brentuximab Vedotin/CD30	Hodgkin lymphoma	FDA (2011) EMA (2012)
Kadcyla^®^	ADC	Trastuzumab emtansine/HER2	Breast cancer	FDA (2013) EMA (2013)
Besponsa^®^	ADC	Inotuzumab ozogamicine/CD22	Acute lymphoblastic leukemia	FDA (2017) EMA (2017)
Yescarta^®^	Adoptive T cell transfer	Axicabtagene ciloleucel/CD19	Lymphoma	FDA (2017)
Kymriah^®^	Adoptive T cell transfer	Tisagenlecleucel/CD19	Acute lymphoblastic leukemia	FDA (2018)
Lumoxiti^®^	ADC	Moxetumomab pasudotox/CD22	Relapsed leukemia	FDA (2018)
Polivy^®^	ADC	Polatuzumab vedotin/CD79	Lymphoma	FDA (2019)
Padcev^®^	ADC	Enfortumab vedotin/Nectin4	Urothelial cancer	FDA (2019)
Enhertu^®^	ADC	Trastuzumab deruxtecan/HER2	Breast cancer	FDA (2020)
Blenrep^®^	ADC	Belantamab mafodotin/BCMA	Myeloma	FDA (2020)
Trodelvy^®^	ADC	Sacituzumab govitecan/TROP2	Breast cancer	FDA (2020)
Zynlonta^®^	ADC	loncastuximab tesirine-lpyl/CD19	Lymphoma	FDA (2021)
Breyanzi^®^	Adoptive T cell transfer	Lisocabtagene maraleucel	Relapsed/refractory large B-cell lymphoma	FDA (2022)

**Table 3:** Anticancer active immunotherapies currently approved by the FDA and/or EMA. * orphan drug designation.

Trade Name	Type of Active Immunotherapies	Drug/Target	Application(s)	Marketing Authorization
Provenge^®^	Therapeutic vaccines	Sipuleucel-T (dendritic cell vaccines)	Prostate cancer	2010
Yervoy^®^	ICI	Ipilimumab/CTLA-4	Melanoma	2011
Opdivo^®^	ICI	Nivolumab/PD-1	Melanoma	2014
			NSCLC	2015
			Kidney cancer	2015
			Hodgkin lymphoma	2016
			Head and neck cancer	2016
			Urothelial cancer	2017
			Hepatocellular carcinoma	2017
			SCLC	2018
			Colorectal cancer	2018
Keytruda^®^	ICI	Pembrolizumab/PD-1	Melanoma	2014
			NSCLC	2015
			Head and neck cancer	2016
			Hodgkin lymphoma	2017
			Urothelial cancer	2017
			Solid metastatic tumors	2017
			Cervix cancer	2018
			PMBCL	2018
			Merkel cell carcinoma	2018
			Kidney cancer	2018
Libtayo^®^	ICI	Cemilplimab/PD-1	Epidermoid carcinoma	2018
Tyvyt^®^	ICI	Sintilimab/PD-1	NSCLC	2021
AiRuiKa^®^	ICI	Camrelizumab/PD-1	Hepatocellular carcinoma	2021 *
Tuoyi^®^	ICI	Toripalimab/PD-1	Esophageal cancer	2021 *
-	ICI	Tislelizumab/PD-1	Esophageal cancer	2021
Bavencio^®^	ICI	Avelumab/PD-L1	Merkel cell carcinoma	2015
			Urothelial cancer	2017
			Kidney cancer	2019
Imfinzi^®^	ICI	Durvalumab/PD-L1	Urothelial cancer	2017
			NSCLC	2018
Tecentriq^®^	ICI	Atezolizumab/PD-L1	Urothelial cancer	2016
			NSCLC	2016
			Breast cancer	2019
Opdualag^®^	ICI	Nivolumab/PD-1 + relatlimab/LAG-3	Metastatic melanoma	2022
Blincyto^®^	BsAbs	Blinatumomab	Lymphoblastic leukemia	2014

**Table 4:** Summary of the most relevant clinical trials in oncology evaluating nanoparticles grafted with antibodies or fragment of antibodies. Adapted from Richards et al. [98].

Name	Type of Nanotherapeutics	Type of Antibody	Target	Drug	Application(s)	Clinical Phase
C225-ILS-Dox	Liposome	Cetuximab fab	EGFR	Doxorubicin	Solid tumors	I (completed in 2020)
Erbitux-EDVspac	Nanocells	Bispecific antibody	EGFR	Paclitaxel	Solid tumors	II
TargomiRs	Nanocells	Bispecific antibody	EGFR	microRNA16a	Mesothelioma	I (completed in 2016)
EGFR(V)-EDV-Dox	Nanocells	Bispecific antibody	EGFR	Doxorubicin + microRNA16a	Glioblastoma	I (ongoing)
*E-EDV-D682*	Nanocells	Bispecific antibody	EGFR	nemorubicin	NSCLC Mesothelioma	I (ongoing)
*E-EDV-D682*	Nanocells	Bispecific antibody	EGFR	nemorubicin	Pancreatic cancer Solid tumors	I/IIa (ongoing)
MM-302	Liposome	Anti-Her2 ScFv	Her2	Doxorubicin	Breast cancer	II (failed in 2018)
Lipovaxin-MM	Liposome	dAb	Dendritic cell CD209	Melanoma antigens + IFNγ	Melanoma vaccine	I (completed in 2012)
MCC-465	Liposome	Anti-GAH F(ab’)_2_	EGFR	Doxorubicin	Metastatic stomach cancer	I (completed 2004)

**Table 5:** Summary of the most relevant current clinical trials in oncology evaluating the association of nab-paclitaxel with ICIs. Previous trials can be found in Soliman et al.’s review [108].

Associated ICI	Application	Clinical Phase	Reference
Nivolumab	Muscle-invasive bladder cancer	II (ongoing)	NCT04876313
	Metastatic Head-and-neck Squamous-cell Carcinoma	II (ongoing)	NCT04831320
	HPV-Related Squamous Cell Carcinoma	II (ongoing)	NCT03107182
	Solid tumors	I/II (ongoing)	NCT04143711
	Nonsmall Cell Lung Cancer	I (ongoing)	NCT04699721
		II (ongoing)	NCT04623775
Pembrolizumab	Nonsmall cell lung cancer	II (completed 2021)	NCT02684461
		I/II (ongoing)	NCT03138889
		I (ongoing)	NCT04297605
		III (ongoing)	NCT03875092, NCT03520686, NCT02775435
	Malignant Neoplasm of Breast	II (completed 2021)	NCT03289819
	Breast Neoplasms	III (ongoing)	NCT04895358
	Urothelial Carcinoma	II (ongoing)	NCT03240016
		II (completed 2020)	NCT03464734
	Hormone-receptor-positive-breast-cancer	I (ongoing)	NCT02999477
	Head and Neck Squamous Cell Carcinoma	II (ongoing)	NCT04857164
	Solid Tumor	I (ongoing)	NCT05017012
	Metastatic-Triple-Negative-Breast-Cancer	II (ongoing)	NCT05174832
	Lung Cancer, Brain Cancer, Cancer	II (ongoing)	NCT04964960
Atezolizumab	Solid Tumor	I (ongoing)	NCT05092373
	Carcinoma	II (ongoing)	NCT03181100
	Triple-Negative Breast Cancer	III (ongoing)	NCT04148911
		II (ongoing)	NCT03961698
		Early Phase 1	NCT04249167
	Renal Cell Carcinoma	II (ongoing)	NCT03961698
Sintilimab	Oropharyngeal Squamous Cell Carcinoma	II (ongoing)	NCT05098119
	Advanced Gastric and Gastro-esophageal Junction Adenocarcinoma	II (ongoing)	NCT04140318, NCT04267549
		I/II (ongoing)	NCT04781413
		II/III (ongoing)	NCT05002686
	Esophageal Squamous Cell Carcinoma	II (ongoing)	NCT04548440
	Breast Cancer	II (ongoing)	NCT04722718
	Head and Neck Squamous Cell Cancer	II (completed 2021)	NCT03975270
	Nonsmall Cell Lung Cancer	III (ongoing)	NCT04840290, NCT05116462
		II (ongoing)	NCT04459611, NCT04846452
		II (ongoing)	NCT04326153
Durvalumab	Head and Neck Squamous Cell Carcinoma	II (ongoing)	NCT03174275
	Pancreatic Cancer	II (ongoing)	NCT04940286
	Nonsmall Cell Lung Cancer	I (ongoing)	NCT05157542, NCT04646837
		I/II (ongoing)	NCT04646837
		II (ongoing)	NCT04905316
		III (ongoing)	NCT03164616
	Breast Cancer	II (ongoing)	NCT03606967
		I/II (ongoing)	NCT04711824
Ipilimumab	Advanced Nonsmall Cell Lung Cancer	II/III (ongoing)	NCT04929041
Camrelizumab	Metastatic Pancreatic Cancer	II (ongoing)	NCT04498689
	Advanced Gastric Cancer	I/II (ongoing)	NCT04286711, NCT05101616
		II (ongoing)	NCT04675866, NCT04258644
	Nonsmall Cell Lung Cancer	II (ongoing)	NCT04167774, NCT04530227, NCT04828395
		II (completed 2021)	NCT04108013, NCT04338620
		III (ongoing)	NCT04768075
	Soft Tissue Sarcoma	II (ongoing)	NCT05189483
	Pancreatic Cancer Stage IV	I (ongoing)	NCT04181645
		III (ongoing)	NCT04674956
	Esophageal Squamous Cell Carcinoma	II (ongoing)	NCT04767295
		I/II (ongoing)	NCT04506138
		II (completed 2021)	NCT04225364
	Adenocarcinoma of the Lung	II (ongoing)	NCT04459078
	Head and Neck Cancer/ Squamous Cell Carcinoma	II (ongoing)	NCT05189184, NCT04922450, NCT04826679
	Cervical Carcinoma	II (completed 2021)	NCT04188860
		II (ongoing)	NCT04680988, NCT04884906
	Thoracic Esophageal Squamous Cell Carcinoma	II (ongoing)	NCT04937673
	Triple-negative Breast Cancer	I/II (ongoing)	NCT04213898
		II (ongoing)	NCT04129996, NCT04537286
	Melanoma	II (ongoing)	NCT04979585
Toripalimab	Advanced Biliary Tract Cancer	II (completed 2021)	NCT04027764
	Urothelial Carcinoma	II (ongoing)	NCT04211012
	Esophagus Cancer	II (ongoing)	NCT04177875, NCT04084158, NCT04844385
	Pancreatic cancer	II (ongoing)	NCT04718701
	Nasopharyngeal Neoplasms	II (ongoing)	NCT04446663
	Gastric Carcinoma	II (ongoing)	NCT04443036
	Oral Squamous Cell Carcinoma	II/III (ongoing)	NCT05125055
		II (ongoing)	NCT04888403, NCT05173246
	Triple Negative Breast Cancer	III (completed 2020)	NCT03777579
		II (completed 2021)	NCT04418154
	Nonsmall Cell Lung Cancer	II (ongoing)	NCT04304248, NCT04725448
Magrolimab	metastatic triple-negative breast cancer	II (ongoing)	NCT04958785
ZKAB001	Advanced urothelial carcinoma	I (ongoing)	NCT04603846
SHR-1701	Nasopharyngeal Carcinoma	I (ongoing)	NCT04282070
Tiragolumab	Triple-Negative Breast Cancer	Ib (ongoing)	NCT04584112
Tislelizumab	Nonmuscle Invasive Bladder Urothelial Carcinoma	II (ongoing)	NCT04730232
	Esophagus Cancer	II (ongoing)	NCT04821765
	Lung Adenocarcinoma Stage IV	II (completed 2021)	NCT04310943
	Locally Advanced and Metastatic Solid Tumors	I (ongoing)	NCT04047862
	Triple-negative Breast Cancer	II (ongoing)	NCT04914390
	Ovarian Cancer	II (ongoing)	NCT04815408
	Muscle-invasive Bladder Cancer	II (ongoing)	NCT04730219

**Table 6:** Summary of the most relevant clinical trials in oncology evaluating nano-immunotherapies’ potential (excluding nanoparticles engrafted with antibodies, and nab-paclitaxel association). * Immunotherapies are given as a different entity, meaning that the immunotherapy is not merged within the nanotherapeutics (i.e., neither linked nor encapsulated).

Name	Association/Merger		Application	Clinical Phase	Reference
	Nanotherapeutics	Immunotherapy			
-	Liposomal doxorubicin	Nivolumab * ipilimumab *	Breast cancer	II	NCT03409198
		Atezolizumab *	Breast cancer	II	NCT03164993
			Ovarian cancer	II/III	NCT02839707
		Pembrolizumab *	Ovarian cancer	I	NCT03596281
			Solid tumors	I	NCT04244552
				II	NCT03539328
		Camrelizumab *	Breast cancer	II	NCT05097248
-	Liposomal irinotecan	Nivolumab *	Biliary Tract Cancer	I/II	NCT03785873
-	Radio-enhancer crystalline hafnium oxide	Nivolumab * pembrolizumab *	Advanced cancers	I	NCT03589339
-	Belantamab mafodotin (ADC)	T cell costimulatory receptor agonist *, Dostarlimab *	Multiple Myeloma	I/II	NCT04126200
-	Brentuximab vedotin (ADC)	Nivolumab *	Hodgkin Lymphoma	II	NCT0275871, NCT03057795, NCT03712202, NCT01716806, NCT01703949
		Nivolumab * + ipilimumab *	Hodgkin Lymphoma	I/II	NCT01896999
-	Trastuzumab deruxtecan (ADC)	Nivolumab *	Breast cancer or urothelial carcinoma	I	NCT03523572
		Pembrolizumab *	Breast cancer or NSCLC	I	NCT04042701
-	Enfortumab vedotin (ADC)	Pembrolizumab *	Urothelial carcinoma	I/II	NCT03288545
BNT111	RNA-lipoplex	Vaccine antigen-specific CD8+/CD4+ T cell + Cemiplimab *	Melanoma	II	NCT04526899
BNT112			Prostate cancer	I/II	NCT04382898
BNT113		Vaccine antigen-specific CD8+/CD4+ T cell + pembrolizumab *	Head and neck cancer	II	NCT04534205
BNT115		Vaccine antigen-specific CD8+/CD4+ T cell	Ovarian cancer	I	NCT04163094
BNT122		patient-specific mRNA vaccine	Colorectal cancer	II	NCT04486378
		patient-specific mRNA vaccine + pembrolizumab *	Advanced Melanoma	II	NCT03815058
		patient-specific mRNA vaccine + atezolizumab *	Solid tumors	I	NCT03289962
			Pancreatic cancer		NCT04161755
BNT141	Lipid-based nanoparticle	mRNA-encoded antibodies	Solid tumors	I/II	NCT04683939
BNT151		mRNA-encoded cytokines			NCT04455620
Oncoquest-L	Liposome	Patient cells + IL-2	Follicular lymphoma	II	NCT02194751
DPX-survivac	Lipid-based nanoparticle	Survivin vaccine + pembrolizumab*	Solid tumors	II	NCT03029403, NCT03836352
			lymphoma		NCT04920617, NCT03349450
DPX-E7	Lipid-based nanoparticle	E7-peptide vaccine	Head and neck, cervix, and anus cancer	I/II	NCT02865135
WDVAX	Polymeric nanoparticle	GM-CSF and CpG + patient cells	Melanoma	I	NCT01753089
mRNA-4157	Lipid-based nanoparticle	mRNA vaccine + pembrolizumab *	Solid tumors	I	NCT03313778
			Melanoma	II	NCT03897881
mRNA-5671			KRAS Mutant Advanced or Metastatic Nonsmall Cell Lung Cancer, Colorectal Cancer, or Pancreatic Adenocarcinoma	I	NCT03948763
GRT-C901/ GRT-C902	Adenoviral and lipidic-based nanoparticles	patient-specific neoantigen cancer vaccine prime/boost + nivolumab */ipilimumab *	Metastatic nonsmall cell lung cancer, microsatellite stable colorectal cancer, gastroesophageal adenocarcinoma, and metastatic urothelial cancer	I/II	NCT03639714
RPTR147	Nanogel	IL-15 Loaded T-Cells + pembrolizumab*	Solid tumors, lymphoma	I	NCT03815682
RiMO 301	Metal organic nanoparticle	indoleamine 2,3-dioxygenase	Advanced tumors	I	NCT03444714
NKTR 214	PEG conjugated	IL-2 linked + anti-PD-1 *	Head and neck Cancer	II	NCT04936841
		IL-2 linked + nivolumab * /ipilimumab *	Solid tumors	I/II	NCT02983045
		IL-2 linked + nivolumab *	Sarcoma	II	NCT03282344
			Melanoma	III	NCT03635983
MEDI1191	Lipid-based nanoparticle	IL-2 linked + durvalumab *	Solid tumors	I	NCT03946800
mRNA-2752		OX40L T cell co-stimulator, IL-23 and IL-36γ pro-inflammatory cytokines + durvalumab*	Solid Tumor Malignancies or Lymphoma	I	NCT03739931
		OX40L T cell co-stimulator, IL-23 and IL-36γ pro-inflammatory cytokines + pembrolizumab *	Carcinoma	I	NCT02872025
Mirzotamab clezutoclax (ABBV-155)	ADC	B7H3 immunodulatory targeted + B cell inhibitory agent	Advanced solid tumors	I	NCT03595059

## References

[1] Tsimberidou A.M., Fountzilas E., Nikanjam M., Kurzrock R. (2020). Review of Precision Cancer Medicine: Evolution of the Treatment Paradigm. Cancer Treat. Rev..

[2] Rodallec A., Benzekry S., Lacarelle B., Ciccolini J., Fanciullino R. (2018). Pharmacokinetics Variability: Why Nanoparticles Are Not Just Magic-Bullets in Oncology. Crit. Rev. Oncol. Hematol..

[3] Yu H., De Geest B.G. (2020). Nanomedicine and Cancer Immunotherapy. Acta Pharm. Sin..

[4] He H., Liu L., Morin E.E., Liu M., Schwendeman A. (2019). Survey of Clinical Translation of Cancer Nanomedicines—Lessons Learned from Successes and Failures. Acc. Chem. Res..

[5] Anselmo A.C., Mitragotri S. (2019). Nanoparticles in the Clinic: An Update. Bioeng. Transl. Med..

[6] Rodallec A., Sicard G., Fanciullino R., Benzekry S., Lacarelle B., Milano G., Ciccolini J. (2018). Turning Cold Tumors into Hot Tumors: Harnessing the Potential of Tumor Immunity Using Nanoparticles. Expert Opin. Drug Metab. Toxicol..

[7] Nakhoda S.K., Olszanski A.J. (2020). Addressing Recent Failures in Immuno-Oncology Trials to Guide Novel Immunotherapeutic Treatment Strategies. Pharm. Med..

[8] Jagodinsky J.C., Harari P.M., Morris Z.S. (2020). The Promise of Combining Radiation Therapy with Immunotherapy. Int. J. Radiat. Oncol. Biol. Phys..

[9] Drake C.G. (2012). Combination Immunotherapy Approaches. Ann. Oncol..

[10] Henricks L.M., Schellens J.H.M., Huitema A.D.R., Beijnen J.H. (2015). The Use of Combinations of Monoclonal Antibodies in Clinical Oncology. Cancer Treat. Rev..

[11] Kaneno R., Shurin G.V., Tourkova I.L., Shurin M.R. (2009). Chemomodulation of Human Dendritic Cell Function by Antineoplastic Agents in Low Noncytotoxic Concentrations. J. Transl. Med..

[12] Shurin G.V., Tourkova I.L., Kaneno R., Shurin M.R. (2009). Chemotherapeutic Agents in Noncytotoxic Concentrations Increase Antigen Presentation by Dendritic Cells via an IL-12-Dependent Mechanism. J. Immunol..

[13] Del Re M., Vivaldi C., Rofi E., Salani F., Crucitta S., Catanese S., Fontanelli L., Massa V., Cucchiara F., Fornaro L. (2021). Gemcitabine Plus Nab-Paclitaxel Induces PD-L1 MRNA Expression in Plasma-Derived Microvesicles in Pancreatic Cancer. Cancers.

[14] Ediriwickrema A., Saltzman W.M. (2015). Nanotherapy for Cancer: Targeting and Multifunctionality in the Future of Cancer Therapies. ACS Biomater. Sci. Eng..

[15] Rodallec A., Fanciullino R., Lacarelle B., Ciccolini J. (2018). Seek and Destroy: Improving PK/PD Profiles of Anticancer Agents with Nanoparticles. Expert Rev. Clin. Pharmacol..

[16] Shi J., Kantoff P.W., Wooster R., Farokhzad O.C. (2017). Cancer Nanomedicine: Progress, Challenges and Opportunities. Nat. Rev. Cancer.

[17] O’Brien M.E.R., Wigler N., Inbar M., Rosso R., Grischke E., Santoro A., Catane R., Kieback D.G., Tomczak P., Ackland S.P. (2004). Reduced Cardiotoxicity and Comparable Efficacy in a Phase III Trial of Pegylated Liposomal Doxorubicin HCl (CAELYX/Doxil) versus Conventional Doxorubicin for First-Line Treatment of Metastatic Breast Cancer. Ann. Oncol..

[18] Von Hoff D.D., Ervin T., Arena F.P., Chiorean E.G., Infante J., Moore M., Seay T., Tjulandin S.A., Ma W.W., Saleh M.N. (2013). Increased Survival in Pancreatic Cancer with Nab-Paclitaxel plus Gemcitabine. N. Engl. J. Med..

[19] Lancet J.E., Uy G.L., Cortes J.E., Newell L.F., Lin T.L., Ritchie E.K., Stuart R.K., Strickland S.A., Hogge D., Solomon S.R. (2018). CPX-351 (Cytarabine and Daunorubicin) Liposome for Injection Versus Conventional Cytarabine Plus Daunorubicin in Older Patients with Newly Diagnosed Secondary Acute Myeloid Leukemia. J. Clin. Oncol..

[20] Donnette M., Hamimed M., Ciccolini J., Berda-Haddad Y., Kaspi E., Venton G., Lacarelle B., Costello R., Ouafik L., Farnault L. (2021). Pharmacokinetics and Pharmacogenetics of Liposomal Cytarabine in AML Patients Treated with CPX-351. J. Control. Release.

[21] Galluzzi L., Vacchelli E., Bravo-San Pedro J.-M., Buqué A., Senovilla L., Baracco E.E., Bloy N., Castoldi F., Abastado J.-P., Agostinis P. (2014). Classification of Current Anticancer Immunotherapies. Oncotarget.

[22] Papaioannou N.E., Beniata O.V., Vitsos P., Tsitsilonis O., Samara P. (2016). Harnessing the Immune System to Improve Cancer Therapy. Ann. Transl. Med..

[23] Wold E.D., Smider V.V., Felding B.H. (2016). Antibody Therapeutics in Oncology. Immunotherapy.

[24] Serna-Gallegos T.R., La-Fargue C.J., Tewari K.S. (2018). The Ecstacy of Gold: Patent Expirations for Trastuzumab, Bevacizumab, Rituximab, and Cetuximab. Recent Pat. Biotechnol..

[25] Slamon D.J., Leyland-Jones B., Shak S., Fuchs H., Paton V., Bajamonde A., Fleming T., Eiermann W., Wolter J., Pegram M. (2001). Use of Chemotherapy plus a Monoclonal Antibody against HER2 for Metastatic Breast Cancer That Overexpresses HER2. N. Engl. J. Med..

[26] Hedrich W.D., Fandy T.E., Ashour H.M., Wang H., Hassan H.E. (2018). Antibody-Drug Conjugates: Pharmacokinetic/Pharmacodynamic Modeling, Preclinical Characterization, Clinical Studies, and Lessons Learned. Clin. Pharmacokinet..

[27] Hafeez U., Parakh S., Gan H.K., Scott A.M. (2020). Antibody-Drug Conjugates for Cancer Therapy. Molecules.

[28] Cortés J., Kim S., Chung W., Im S., Park Y.H., Hegg R., Kim M.H., Tseng L., Petry V., Chung C. (2021). LBA1-Trastuzumab Deruxtecan (T-DXd) vs Trastuzumab Emtansine (T-DM1) in Patients (Pts) with HER2+ Metastatic Breast Cancer (MBC): Results of the Randomized Phase III DESTINY-Breast03 Study. Ann. Oncol..

[29] Modi S., Jacot W., Yamashita T., Sohn J., Vidal M., Tokunaga E., Tsurutani J., Ueno N.T., Chae Y.S., Lee K.S. (2022). Trastuzumab Deruxtecan (T-DXd) versus Treatment of Physician’s Choice (TPC) in Patients (Pts) with HER2-Low Unresectable and/or Metastatic Breast Cancer (MBC): Results of DESTINY-Breast04, a Randomized, Phase 3 Study. JCO.

[30] Wang Z., Cao Y.J. (2020). Adoptive Cell Therapy Targeting Neoantigens: A Frontier for Cancer Research. Front. Immunol..

[31] Kalos M., June C.H. (2013). Adoptive T Cell Transfer for Cancer Immunotherapy in the Era of Synthetic Biology. Immunity.

[32] Wang Z., Wu Z., Liu Y., Han W. (2017). New Development in CAR-T Cell Therapy. J. Hematol. Oncol..

[33] Titov A., Kaminskiy Y., Ganeeva I., Zmievskaya E., Valiullina A., Rakhmatullina A., Petukhov A., Miftakhova R., Rizvanov A., Bulatov E. (2022). Knowns and Unknowns about CAR-T Cell Dysfunction. Cancers.

[34] Darvin P., Toor S.M., Sasidharan Nair V., Elkord E. (2018). Immune Checkpoint Inhibitors: Recent Progress and Potential Biomarkers. Exp. Mol. Med..

[35] Larkin J., Chiarion-Sileni V., Gonzalez R., Grob J.J., Cowey C.L., Lao C.D., Schadendorf D., Dummer R., Smylie M., Rutkowski P. (2015). Combined Nivolumab and Ipilimumab or Monotherapy in Untreated Melanoma. N. Engl. J. Med..

[36] Robert C., Schachter J., Long G.V., Arance A., Grob J.J., Mortier L., Daud A., Carlino M.S., McNeil C., Lotem M. (2015). Pembrolizumab versus Ipilimumab in Advanced Melanoma. N. Engl. J. Med..

[37] Keam S.J. (2019). Toripalimab: First Global Approval. Drugs.

[38] Markham A., Keam S.J. (2019). Camrelizumab: First Global Approval. Drugs.

[39] Lee A., Keam S.J. (2020). Tislelizumab: First Approval. Drugs.

[40] Sezer A., Kilickap S., Gümüş M., Bondarenko I., Özgüroğlu M., Gogishvili M., Turk H.M., Cicin I., Bentsion D., Gladkov O. (2021). Cemiplimab Monotherapy for First-Line Treatment of Advanced Non-Small-Cell Lung Cancer with PD-L1 of at Least 50%: A Multicentre, Open-Label, Global, Phase 3, Randomised, Controlled Trial. Lancet.

[41] Francisco L.M., Salinas V.H., Brown K.E., Vanguri V.K., Freeman G.J., Kuchroo V.K., Sharpe A.H. (2009). PD-L1 Regulates the Development, Maintenance, and Function of Induced Regulatory T Cells. J. Exp. Med..

[42] Cao Y., Wang X., Jin T., Tian Y., Dai C., Widarma C., Song R., Xu F. (2020). Immune Checkpoint Molecules in Natural Killer Cells as Potential Targets for Cancer Immunotherapy. Signal Transduct. Target. Ther..

[43] Tawbi H.A., Schadendorf D., Lipson E.J., Ascierto P.A., Matamala L., Castillo Gutiérrez E., Rutkowski P., Gogas H.J., Lao C.D., De Menezes J.J. (2022). Relatlimab and Nivolumab versus Nivolumab in Untreated Advanced Melanoma. N. Engl. J. Med..

[44] Ma J., Mo Y., Tang M., Shen J., Qi Y., Zhao W., Huang Y., Xu Y., Qian C. (2021). Bispecific Antibodies: From Research to Clinical Application. Front. Immunol..

[45] Schwartz M.S., Jeyakumar D., Damon L.E., Schiller G.J., Wieduwilt M.J. (2019). A Phase I/II Study of Blinatumomab in Combination with Pembrolizumab for Adults with Relapsed Refractory B-Lineage Acute Lymphoblastic Leukemia: University of California Hematologic Malignancies Consortium Study 1504. JCO.

[46] Sandhu K.S., Huynh-Tran Q., Cooper E.E., zhang J., Palmer J., Tsai N.-C., Thomas S., Robbins M., Aribi A., Salhotra A. (2021). ALL-440: Promising Safety and Efficacy Results from an Ongoing Phase 1/2 Study of Pembrolizumab in Combination with Blinatumomab in Patients (Pts) with Relapsed or Refractory (R/R) Acute Lymphoblastic Leukemia (ALL). Clin. Lymphoma Myeloma Leuk..

[47] Giri P., Patil S., Ratnasingam S., Prince H.M., Milliken S., Briones Meijide J., Coyle L., Van Der Poel M., Mulroney C.M., Farooqui M.Z.H. (2022). Results from a Phase 1b Study of Blinatumomab-Pembrolizumab Combination in Adults with Relapsed/Refractory (R/R) Diffuse Large B-Cell Lymphoma (DLBCL). JCO.

[48] Wang J., Mamuti M., Wang H. (2020). Therapeutic Vaccines for Cancer Immunotherapy. ACS Biomater. Sci. Eng..

[49] Song Q., Zhang C.-D., Wu X.-H. (2018). Therapeutic Cancer Vaccines: From Initial Findings to Prospects. Immunol. Lett..

[50] Kantoff P.W., Higano C.S., Shore N.D., Berger E.R., Small E.J., Penson D.F., Redfern C.H., Ferrari A.C., Dreicer R., Sims R.B. (2010). Sipuleucel-T Immunotherapy for Castration-Resistant Prostate Cancer. N. Engl. J. Med..

[51] Guevara M.L., Persano F., Persano S. (2021). Nano-Immunotherapy: Overcoming Tumour Immune Evasion. Semin. Cancer Biol..

[52] Maeda H. (2001). The Enhanced Permeability and Retention (EPR) Effect in Tumor Vasculature: The Key Role of Tumor-Selective Macromolecular Drug Targeting. Adv. Enzym. Regul..

[53] Fanciullino R., Mollard S., Correard F., Giacometti S., Serdjebi C., Iliadis A., Ciccolini J. (2014). Biodistribution, Tumor Uptake and Efficacy of 5-FU-Loaded Liposomes: Why Size Matters. Pharm. Res..

[54] Charrois G.J.R., Allen T.M. (2003). Rate of Biodistribution of STEALTH® Liposomes to Tumor and Skin: Influence of Liposome Diameter and Implications for Toxicity and Therapeutic Activity. Biochim. Et Biophys. Acta (BBA)-Biomembr..

[55] Hare J.I., Lammers T., Ashford M.B., Puri S., Storm G., Barry S.T. (2017). Challenges and Strategies in Anti-Cancer Nanomedicine Development: An Industry Perspective. Adv. Drug Deliv. Rev..

[56] Eerden R.A.G.V., Atrafi F., vanHylckama Vlieg M.A.M., Hoop E.O., de Bruijn P., Moelker A., Lolkema M.P., Rijcken C.J.F., Hanssen R., Eskens F.A. (2019). Comparison of Intratumoral Docetaxel Exposure in Cancer Patients between Nanoparticle Entrapped Docetaxel (CPC634) and Conventional Docetaxel (Cd): The CriTax Study. Ann. Oncol..

[57] Wilhelm S., Tavares A.J., Dai Q., Ohta S., Audet J., Dvorak H.F., Chan W.C.W. (2016). Analysis of Nanoparticle Delivery to Tumours. Nat. Rev. Mater..

[58] Park J., Choi Y., Chang H., Um W., Ryu J.H., Kwon I.C. (2019). Alliance with EPR Effect: Combined Strategies to Improve the EPR Effect in the Tumor Microenvironment. Theranostics.

[59] Sorace A.G., Quarles C.C., Whisenant J.G., Hanker A.B., McIntyre J.O., Sanchez V.M., Yankeelov T.E. (2016). Trastuzumab Improves Tumor Perfusion and Vascular Delivery of Cytotoxic Therapy in a Murine Model of HER2+ Breast Cancer: Preliminary Results. Breast Cancer Res. Treat..

[60] Sicard G., Rodallec A., Correard F., Vaghi C., Poignard C., Ciccolini J., Benzekry S., Sergé A., Fanciullino R. (2020). Abstract 6244: Turning Poorly Vascularized Tumors into Highly Vascularized Tumors with Nanoparticles: Proof of Concept and Pharmacometric Analysis. Cancer Res..

[61] Di J., Xie F., Xu Y. (2020). When Liposomes Met Antibodies: Drug Delivery and Beyond. Adv. Drug Deliv. Rev..

[62] Rodallec A., Sicard G., Giacometti S., Carré M., Pourroy B., Bouquet F., Savina A., Lacarelle B., Ciccolini J., Fanciullino R. (2018). From 3D Spheroids to Tumor Bearing Mice: Efficacy and Distribution Studies of Trastuzumab-Docetaxel Immunoliposome in Breast Cancer. Int. J. Nanomed..

[63] Kirpotin D.B., Drummond D.C., Shao Y., Shalaby M.R., Hong K., Nielsen U.B., Marks J.D., Benz C.C., Park J.W. (2006). Antibody Targeting of Long-Circulating Lipidic Nanoparticles Does Not Increase Tumor Localization but Does Increase Internalization in Animal Models. Cancer Res..

[64] Kao H.-W., Lin Y.-Y., Chen C.-C., Chi K.-H., Tien D.-C., Hsia C.-C., Lin W.-J., Chen F.-D., Lin M.-H., Wang H.-E. (2014). Biological Characterization of Cetuximab-Conjugated Gold Nanoparticles in a Tumor Animal Model. Nanotechnology.

[65] Bazak R., Houri M., El Achy S., Kamel S., Refaat T. (2015). Cancer Active Targeting by Nanoparticles: A Comprehensive Review of Literature. J. Cancer Res. Clin. Oncol..

[66] Xu S., Cui F., Huang D., Zhang D., Zhu A., Sun X., Cao Y., Ding S., Wang Y., Gao E. (2019). PD-L1 Monoclonal Antibody-Conjugated Nanoparticles Enhance Drug Delivery Level and Chemotherapy Efficacy in Gastric Cancer Cells. Int. J. Nanomed..

[67] Sousa F., Castro P., Fonte P., Kennedy P.J., Neves-Petersen M.T., Sarmento B. (2017). Nanoparticles for the Delivery of Therapeutic Antibodies: Dogma or Promising Strategy?. Expert Opin. Drug Deliv..

[68] Lammers T., Kiessling F., Ashford M., Hennink W., Crommelin D., Storm G. (2016). Cancer Nanomedicine: Is Targeting Our Target?. Nat. Rev. Mater..

[69] Ceña V., Játiva P. (2018). Nanoparticle Crossing of Blood–Brain Barrier: A Road to New Therapeutic Approaches to Central Nervous System Diseases. Nanomedicine.

[70] Sousa F., Dhaliwal H.K., Gattacceca F., Sarmento B., Amiji M.M. (2019). Enhanced Anti-Angiogenic Effects of Bevacizumab in Glioblastoma Treatment upon Intranasal Administration in Polymeric Nanoparticles. J. Control. Release.

[71] El Hallal R., Lyu N., Wang Y. (2021). Effect of Cetuximab-Conjugated Gold Nanoparticles on the Cytotoxicity and Phenotypic Evolution of Colorectal Cancer Cells. Molecules.

[72] Chiu G.N.C., Edwards L.A., Kapanen A.I., Malinen M.M., Dragowska W.H., Warburton C., Chikh G.G., Fang K.Y.Y., Tan S., Sy J. (2007). Modulation of Cancer Cell Survival Pathways Using Multivalent Liposomal Therapeutic Antibody Constructs. Mol. Cancer Ther..

[73] Shi Y., Lammers T. (2019). Combining Nanomedicine and Immunotherapy. Acc. Chem. Res..

[74] Nanomedicine and Cancer Immunotherapy | Acta Pharmacologica Sinica. https://www.nature.com/articles/s41401-020-0426-2.

[75] Zhao X., Yang K., Zhao R., Ji T., Wang X., Yang X., Zhang Y., Cheng K., Liu S., Hao J. (2016). Inducing Enhanced Immunogenic Cell Death with Nanocarrier-Based Drug Delivery Systems for Pancreatic Cancer Therapy. Biomaterials.

[76] Rios-Doria J., Durham N., Wetzel L., Rothstein R., Chesebrough J., Holoweckyj N., Zhao W., Leow C.C., Hollingsworth R. (2015). Doxil Synergizes with Cancer Immunotherapies to Enhance Antitumor Responses in Syngeneic Mouse Models. Neoplasia.

[77] Yang S., Sun I.-C., Hwang H.S., Shim M.K., Yoon H.Y., Kim K. (2021). Rediscovery of Nanoparticle-Based Therapeutics: Boosting Immunogenic Cell Death for Potential Application in Cancer Immunotherapy. J. Mater. Chem. B.

[78] Jiang M., Chen W., Yu W., Xu Z., Liu X., Jia Q., Guan X., Zhang W. (2021). Sequentially PH-Responsive Drug-Delivery Nanosystem for Tumor Immunogenic Cell Death and Cooperating with Immune Checkpoint Blockade for Efficient Cancer Chemoimmunotherapy. ACS Appl. Mater. Interfaces.

[79] Hu M., Zhang J., Kong L., Yu Y., Hu Q., Yang T., Wang Y., Tu K., Qiao Q., Qin X. (2021). Immunogenic Hybrid Nanovesicles of Liposomes and Tumor-Derived Nanovesicles for Cancer Immunochemotherapy. ACS Nano.

[80] Baghban R., Roshangar L., Jahanban-Esfahlan R., Seidi K., Ebrahimi-Kalan A., Jaymand M., Kolahian S., Javaheri T., Zare P. (2020). Tumor Microenvironment Complexity and Therapeutic Implications at a Glance. Cell Commun. Signal..

[81] Musetti S., Huang L. (2018). Nanoparticle-Mediated Remodeling of the Tumor Microenvironment to Enhance Immunotherapy. ACS Nano.

[82] Zhang P., Darmon A., Marill J., Mohamed Anesary N., Paris S. (2020). Radiotherapy-Activated Hafnium Oxide Nanoparticles Produce Abscopal Effect in a Mouse Colorectal Cancer Model. Int. J. Nanomed..

[83] Hu Y., Paris S., Barsoumian H., Abana C.O., He K., Sezen D., Wasley M., Masrorpour F., Chen D., Yang L. (2021). A Radioenhancing Nanoparticle Mediated Immunoradiation Improves Survival and Generates Long-Term Antitumor Immune Memory in an Anti-PD1-Resistant Murine Lung Cancer Model. J. Nanobiotechnol..

[84] Walters A.A., Dhadwar B., Al-Jamal K.T. (2021). Modulating Expression of Inhibitory and Stimulatory Immune ‘Checkpoints’ Using Nanoparticulate-Assisted Nucleic Acid Delivery. EBioMedicine.

[85] Esmaily M., Masjedi A., Hallaj S., Nabi Afjadi M., Malakotikhah F., Ghani S., Ahmadi A., Sojoodi M., Hassannia H., Atyabi F. (2020). Blockade of CTLA-4 Increases Anti-Tumor Response Inducing Potential of Dendritic Cell Vaccine. J. Control. Release.

[86] Arend R.C., Beer H.M., Cohen Y.C., Berlin S., Birrer M.J., Campos S.M., Rachmilewitz Minei T., Harats D., Wall J.A., Foxall M.E. (2020). Ofranergene Obadenovec (VB-111) in Platinum-Resistant Ovarian Cancer; Favorable Response Rates in a Phase I/II Study Are Associated with an Immunotherapeutic Effect. Gynecol. Oncol..

[87] Taheri R.A., Bahramifar A., Jaafari M.R., Fasihi-Ramandi M., Emameh R.Z., Mohammadian Haftcheshmeh S., Ebrahimi Nik M. (2021). Designing New Nanoliposomal Formulations and Evaluating Their Effects on Myeloid-Derived Suppressor Cells and Regulatory T Cells in a Colon Cancer Model Aiming to Develop an Efficient Delivery System for Cancer Treatment; an in Vitro and in Vivo Study. Biotechnol. Appl. Biochem..

[88] Lin L., Hu X., Zhang H., Hu H. (2019). Tertiary Lymphoid Organs in Cancer Immunology: Mechanisms and the New Strategy for Immunotherapy. Front. Immunol..

[89] Sun Q., Barz M., De Geest B.G., Diken M., Hennink W.E., Kiessling F., Lammers T., Shi Y. (2019). Nanomedicine and Macroscale Materials in Immuno-Oncology. Chem. Soc. Rev..

[90] Zeng B., Middelberg A.P.J., Gemiarto A., MacDonald K., Baxter A.G., Talekar M., Moi D., Tullett K.M., Caminschi I., Lahoud M.H. (2018). Self-Adjuvanting Nanoemulsion Targeting Dendritic Cell Receptor Clec9A Enables Antigen-Specific Immunotherapy. J. Clin. Investig..

[91] Li S., Feng X., Wang J., Xu W., Islam M.A., Sun T., Xie Z., Wang C., Ding J., Chen X. (2019). Multiantigenic Nanoformulations Activate Anticancer Immunity Depending on Size. Adv. Funct. Mater..

[92] Priem B., van Leent M.M.T., Teunissen A.J.P., Sofias A.M., Mourits V.P., Willemsen L., Klein E.D., Oosterwijk R.S., Meerwaldt A.E., Munitz J. (2020). Trained Immunity-Promoting Nanobiologic Therapy Suppresses Tumor Growth and Potentiates Checkpoint Inhibition. Cell.

[93] Giustarini G., Pavesi A., Adriani G. (2021). Nanoparticle-Based Therapies for Turning Cold Tumors Hot: How to Treat an Immunosuppressive Tumor Microenvironment. Front. Bioeng. Biotechnol..

[94] Sacdalan D.B., Lucero J.A., Sacdalan D.L. (2018). Prognostic Utility of Baseline Neutrophil-to-Lymphocyte Ratio in Patients Receiving Immune Checkpoint Inhibitors: A Review and Meta-Analysis. OncoTargets Ther..

[95] Green M.R., Manikhas G.M., Orlov S., Afanasyev B., Makhson A.M., Bhar P., Hawkins M.J. (2006). Abraxane®, a Novel Cremophor®-Free, Albumin-Bound Particle Form of Paclitaxel for the Treatment of Advanced Non-Small-Cell Lung Cancer. Ann. Oncol..

[96] Giles A.J., Hutchinson M.-K.N.D., Sonnemann H.M., Jung J., Fecci P.E., Ratnam N.M., Zhang W., Song H., Bailey R., Davis D. (2018). Dexamethasone-Induced Immunosuppression: Mechanisms and Implications for Immunotherapy. J. ImmunoTherapy Cancer.

[97] Maxwell R., Luksik A.S., Garzon-Muvdi T., Hung A.L., Kim E.S., Wu A., Xia Y., Belcaid Z., Gorelick N., Choi J. (2018). Contrasting Impact of Corticosteroids on Anti-PD-1 Immunotherapy Efficacy for Tumor Histologies Located within or Outside the Central Nervous System. OncoImmunology.

[98] Richards D.A., Maruani A., Chudasama V. (2017). Antibody Fragments as Nanoparticle Targeting Ligands: A Step in the Right Direction. Chem. Sci..

[99] Brahmbhatt H., MacDiarmid J.A. (2022). Bacterial Minicells to the Rescue: Cyto-Immunotherapy for the Treatment of Late Stage Cancers with Minimal to No Toxicity. Microb. Biotechnol..

[100] van Zandwijk N., Pavlakis N., Kao S.C., Linton A., Boyer M.J., Clarke S., Huynh Y., Chrzanowska A., Fulham M.J., Bailey D.L. (2017). Safety and Activity of MicroRNA-Loaded Minicells in Patients with Recurrent Malignant Pleural Mesothelioma: A First-in-Man, Phase 1, Open-Label, Dose-Escalation Study. Lancet Oncol..

[101] Gargett T., Abbas M.N., Rolan P., Price J.D., Gosling K.M., Ferrante A., Ruszkiewicz A., Atmosukarto I.I.C., Altin J., Parish C.R. (2018). Phase I Trial of Lipovaxin-MM, a Novel Dendritic Cell-Targeted Liposomal Vaccine for Malignant Melanoma. Cancer Immunol. Immunother..

[102] Matsumura Y., Gotoh M., Muro K., Yamada Y., Shirao K., Shimada Y., Okuwa M., Matsumoto S., Miyata Y., Ohkura H. (2004). Phase I and Pharmacokinetic Study of MCC-465, a Doxorubicin (DXR) Encapsulated in PEG Immunoliposome, in Patients with Metastatic Stomach Cancer. Ann. Oncol..

[103] Prokop A., Weissig V. (2016). Intracellular Delivery III: Market Entry Barriers of Nanomedicines.

[104] Pondé N., Aftimos P., Piccart M. (2019). Antibody-Drug Conjugates in Breast Cancer: A Comprehensive Review. Curr. Treat. Options Oncol..

[105] Martínez-Sáez O., Prat A. (2021). Current and Future Management of HER2-Positive Metastatic Breast Cancer. JCO Oncol. Pract..

[106] Schmid P., Adams S., Rugo H.S., Schneeweiss A., Barrios C.H., Iwata H., Diéras V., Hegg R., Im S.-A., Shaw Wright G. (2018). Atezolizumab and Nab-Paclitaxel in Advanced Triple-Negative Breast Cancer. N. Engl. J. Med..

[107] Miles D., Gligorov J., André F., Cameron D., Schneeweiss A., Barrios C., Xu B., Wardley A., Kaen D., Andrade L. (2021). Primary Results from IMpassion131, a Double-Blind, Placebo-Controlled, Randomised Phase III Trial of First-Line Paclitaxel with or without Atezolizumab for Unresectable Locally Advanced/Metastatic Triple-Negative Breast Cancer. Ann. Oncol..

[108] Soliman H.H. (2017). Nab-Paclitaxel as a Potential Partner with Checkpoint Inhibitors in Solid Tumors. OncoTargets Ther..

[109] Mandó P., Rivero S.G., Rizzo M.M., Pinkasz M., Levy E.M. (2021). Targeting ADCC: A Different Approach to HER2 Breast Cancer in the Immunotherapy Era. Breast.

[110] Gerber H.-P., Sapra P., Loganzo F., May C. (2016). Combining Antibody-Drug Conjugates and Immune-Mediated Cancer Therapy: What to Expect?. Biochem. Pharm..

[111] Advani R.H., Moskowitz A.J., Bartlett N.L., Vose J.M., Ramchandren R., Feldman T.A., LaCasce A.S., Christian B.A., Ansell S.M., Moskowitz C.H. (2021). Brentuximab Vedotin in Combination with Nivolumab in Relapsed or Refractory Hodgkin Lymphoma: 3-Year Study Results. Blood.

[112] Drago J.Z., Modi S., Chandarlapaty S. (2021). Unlocking the Potential of Antibody–Drug Conjugates for Cancer Therapy. Nat. Rev. Clin. Oncol..

[113] Briukhovetska D., Dörr J., Endres S., Libby P., Dinarello C.A., Kobold S. (2021). Interleukins in Cancer: From Biology to Therapy. Nat. Rev. Cancer.

[114] Xue H.Y., Guo P., Wen W.-C., Wong H.L. (2015). Lipid-Based Nanocarriers for RNA Delivery. Curr. Pharm. Des..

[115] Dacoba T.G., Anthiya S., Berrecoso G., Fernández-Mariño I., Fernández-Varela C., Crecente-Campo J., Teijeiro-Osorio D., Torres Andón F., Alonso M.J. (2021). Nano-Oncologicals: A Tortoise Trail Reaching New Avenues. Adv. Funct. Mater..

[116] Sahin U., Oehm P., Derhovanessian E., Jabulowsky R.A., Vormehr M., Gold M., Maurus D., Schwarck-Kokarakis D., Kuhn A.N., Omokoko T. (2020). An RNA Vaccine Drives Immunity in Checkpoint-Inhibitor-Treated Melanoma. Nature.

[117] Sun Q., Bai X., Sofias A.M., van der Meel R., Ruiz-Hernandez E., Storm G., Hennink W.E., De Geest B., Kiessling F., Yu H. (2020). Cancer Nanomedicine Meets Immunotherapy: Opportunities and Challenges. Acta Pharm. Sin..

[118] Pujade-Lauraine E., Fujiwara K., Ledermann J.A., Oza A.M., Kristeleit R., Ray-Coquard I.-L., Richardson G.E., Sessa C., Yonemori K., Banerjee S. (2021). Avelumab Alone or in Combination with Chemotherapy versus Chemotherapy Alone in Platinum-Resistant or Platinum-Refractory Ovarian Cancer (JAVELIN Ovarian 200): An Open-Label, Three-Arm, Randomised, Phase 3 Study. Lancet Oncol..

[119] van der Meel R., Sulheim E., Shi Y., Kiessling F., Mulder W.J.M., Lammers T. (2019). Smart Cancer Nanomedicine: Strategic Directions to Improve Translation and Exploitation. Nat. Nanotechnol..

[120] Sankar K., Ye J.C., Li Z., Zheng L., Song W., Hu-Lieskovan S. (2022). The Role of Biomarkers in Personalized Immunotherapy. Biomark. Res..

[121] Zhu S., Zhang T., Zheng L., Liu H., Song W., Liu D., Li Z., Pan C. (2021). Combination Strategies to Maximize the Benefits of Cancer Immunotherapy. J. Hematol. Oncol..

[122] Alimohammadi R., Alibeigi R., Nikpoor A.R., Chalbatani G.M., Webster T.J., Jaafari M.R., Jalali S.A. (2020). Encapsulated Checkpoint Blocker Before Chemotherapy: The Optimal Sequence of Anti-CTLA-4 and Doxil Combination Therapy. Int. J. Nanomed..

[123] Rodallec A., Fanciullino R., Benzekry S., Ciccolini J., EORTC PAMM Group (2019). Is There Any Room for Pharmacometrics with Immuno-Oncology Drugs? Input from the EORTC-PAMM Course on Preclinical and Early-Phase Clinical Pharmacology. Anticancer Res..

[124] Cheng S., Nethi S.K., Al-Kofahi M., Prabha S. (2021). Pharmacokinetic—Pharmacodynamic Modeling of Tumor Targeted Drug Delivery Using Nano-Engineered Mesenchymal Stem Cells. Pharmaceutics.

